# Genotyping bacterial and fungal pathogens using sequence variation in the gene for the CCA-adding enzyme

**DOI:** 10.1186/s12866-016-0670-2

**Published:** 2016-03-18

**Authors:** Paul Franz, Heike Betat, Mario Mörl

**Affiliations:** Institute for Biochemistry, Leipzig University, Brüderstr. 34, 04103, Leipzig, Germany

**Keywords:** Pathogen detection, Infectious diseases, Genotyping, CCA-adding enzyme, tRNA nucleotidyltransferase, Sequence analysis

## Abstract

**Background:**

To allow an immediate treatment of an infection with suitable antibiotics and bactericides or fungicides, there is an urgent need for fast and precise identification of the causative human pathogens. Methods based on DNA sequence comparison like 16S rRNA analysis have become standard tools for pathogen verification. However, the distinction of closely related organisms remains a challenging task. To overcome such limitations, we identified a new genomic target sequence located in the single copy gene for tRNA nucleotidyltransferase fulfilling the requirements for a ubiquitous, yet highly specific DNA marker. In the present study, we demonstrate that this sequence marker has a higher discriminating potential than commonly used genotyping markers in pro- as well as eukaryotes, underscoring its applicability as an excellent diagnostic tool in infectology.

**Results:**

Based on phylogenetic analyses, a region within the gene for tRNA nucleotidyltransferase (CCA-adding enzyme) was identified as highly heterogeneous. As prominent examples for pro- and eukaryotic pathogens, several *Vibrio* and *Aspergillus* species were used for genotyping and identification in a multiplex PCR approach followed by gel electrophoresis and fluorescence-based product detection. Compared to rRNA analysis, the selected gene region of the tRNA nucleotidyltransferase revealed a seven to 30-fold higher distinction potential between closely related *Vibrio* or *Aspergillus* species, respectively. The obtained data exhibit a superb genome specificity in the diagnostic analysis. Even in the presence of a 1,000-fold excess of human genomic DNA, no unspecific amplicons were produced.

**Conclusions:**

These results indicate that a relatively short segment of the coding region for tRNA nucleotidyltransferase has a higher discriminatory potential than most established diagnostic DNA markers. Besides identifying microbial pathogens in infections, further possible applications of this new marker are food hygiene controls or metagenome analyses.

**Electronic supplementary material:**

The online version of this article (doi:10.1186/s12866-016-0670-2) contains supplementary material, which is available to authorized users.

## Background

Bacterial and fungal infections are one of the major threats to human health. Among others, increasing resistances of pathogenic microorganisms against frequently used antibiotics are raising the number of dangerous infections every year [1]. Hence, a fast and reliable identification system to achieve an effective and specific medical treatment is essential [2, 3]. In the last decades, procedures based on morphological, physiological and biochemical analyses were progressively replaced by more sensitive PCR amplifications of pathogen-specific gene sequences. These target sequences exhibit patterns of characteristic point mutations, allowing a robust species-specific verification of an organism [4, 5].

In this study, we present a new and highly specific target sequence for genotyping bacterial and eukaryotic pathogens. The CCA-adding enzyme (ATP(CTP):tRNA nucleotidyltransferase) represents an essential and ubiquitous tRNA maturation activity found in all kingdoms of life. The enzyme catalyzes the posttranscriptional addition of the CCA triplet to the 3′-end of tRNAs, generating the site of aminoacylation [6–8]. Even in organisms where the CCA triplet is encoded in the tRNA genes, like *E. coli* or certain *Bacillus* species, a CCA-adding enzyme is encoded in the genome, as it is also required for the maintenance and repair of the CCA-ends in the tRNA pool [9–11].

**Fig. 1:**
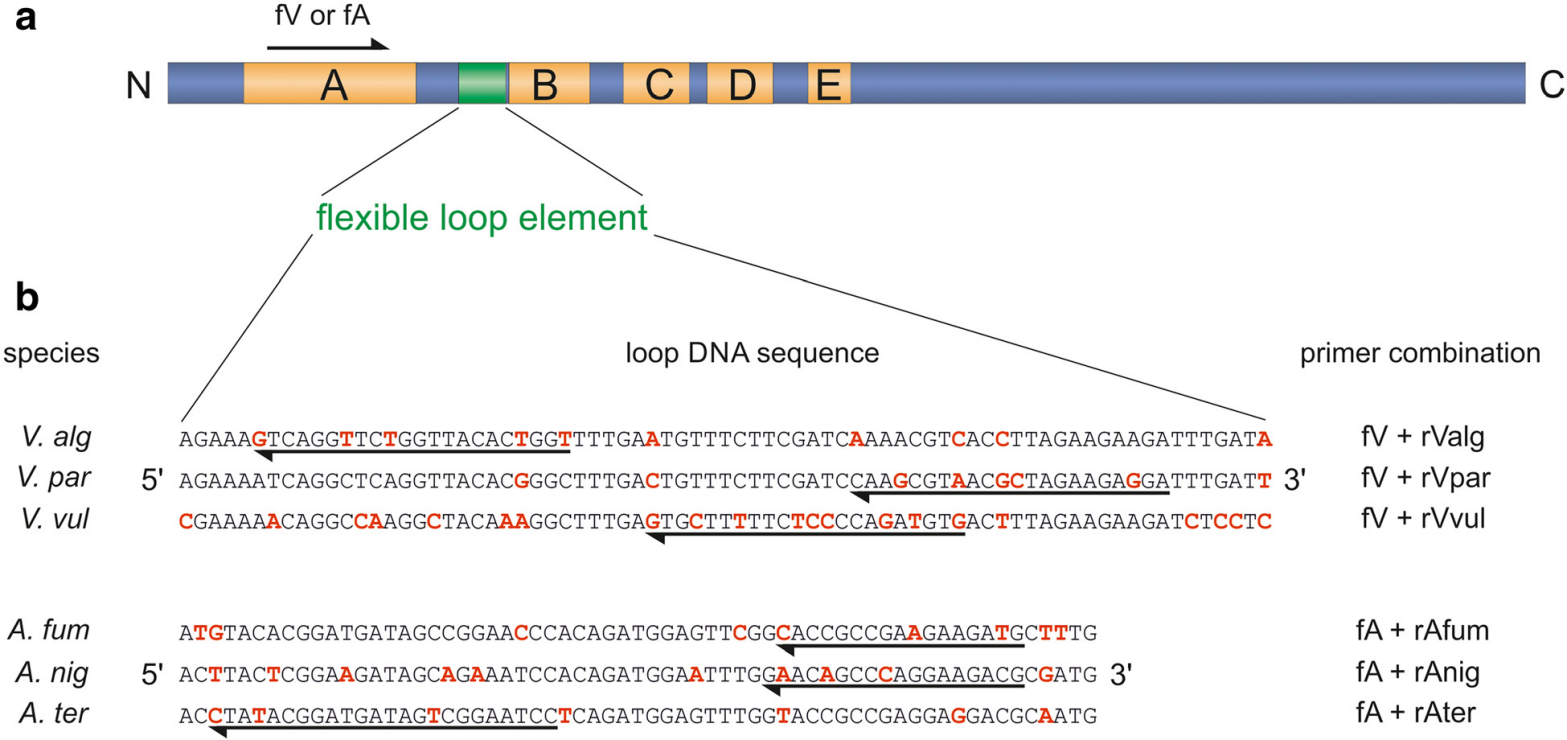
A highly variable loop element in CCA-adding enzymes. **a**. The flexible loop (*green*) is located between two highly conserved motifs A and B of the catalytic core. At the DNA level, this allows for the annealing of a genus-specific forward primer fV (*Vibrio*) or fA (*Aspergillus*), indicated by the *arrow*. **b**. Sequence alignment of the individual loop regions of *V. alginolyticus*, *V. parahaemolyticus*, *V. vulnificus*, *A. fumigatus*, *A.* *niger*, and *A. terreus*. *Red* characters indicate species-specific point mutations. *Arrows* indicate the hybridizing regions of the reverse primers. Primer combinations used in the amplification reactions are shown on the *right*

Bacterial and eukaryotic CCA-adding enzymes share five highly conserved sequence motifs A to E in the N-terminal catalytic core (Fig. 1a) [12]. Molecular modelling as well as crystal structures indicate that a sequence stretch of 10 to 25 amino acids between motifs A and B forms a flexible loop element that, according to biochemical characterization, is involved in the CCA polymerization reaction [12–15]. Despite this important function, the flexible loop region follows a highly unusual evolutionary path differing from that of the other core motifs. While motifs A to E show a high conservation at the amino acid level, the sequence of the loop element varies dramatically between different organisms. Accordingly, the protein as well as DNA sequence pattern of this region allows for a classification into different sequence families that perfectly represent individual phylogenetic genera [15]. Here, we show that this species-specific sequence composition fulfils the criteria for a reliable new genotyping marker for pro- as well as eukaryotic causative organisms. As a proof of principle, we demonstrate that the loop-encoding DNA region is suited to identify and discriminate even very closely related pathogenic strains in the groups of *Vibrio* and *Aspergillus*.

Besides *V. cholerae*, other *Vibrio* species represent highly pathogenic germs as well. *V. alginolyticus*, *V. parahaemolyticus* and *V. vulnificus* are gram-negative, halophilic bacteria that are frequently found in estuarine, coastal and other nutrient-rich waters around the world [16–22]. They are frequently detected in seafood like oysters, shrimps or soft-shell clams. Consequently, they are associated with gastroenteritis and septicemia as well as wound infections due to direct contact with contaminated water [23–31]. Studies in the United States show a correlation between *V. parahaemolyticus* counts and the number of zooplankton populations, which are increasing during the summer months, when the incidence for *Vibrio* infections is high [32–34]. In cooler seasons, *Vibrio* species only occur in sediments or shellfish before they start to proliferate again at warmer temperatures with one of the fastest bacterial growth rates [32, 35–38]. Accordingly, increased surface temperatures of lakes and seas correlate with an elevated infection incidence [39–43].

In addition to these examples of closely related prokaryotic pathogens, we have included several species of *Aspergillus*, representing a eukaryotic disease-causing fungus. The most prominent pathogenic species of this mold genus are *A. fumigatus*, *A. niger* and *A. terreus,* triggering life-threatening invasive infections which are a leading cause of mortality in immunocompromised patients [44–49]. *A. fumigatus* is the main causative agent of invasive aspergillosis that affects skin, ears, paranasal sinuses or the lung, resulting in a mortality rate of nearly 100 % in untreated patients [44]. Infections with *A. niger* and *A. terreus* also frequently cause serious aspergillosis with comparable mortality rates [44]. These infections represent a worldwide health threat, as several studies show a ubiquitous presence of *Aspergillus* species. In addition to soil samples and internal building walls, they can also be isolated from clothes, shower heads, dusty air conditioners and hospital plants, resulting in a constantly high infection incidence in immunocompromised patients [48, 50].

Thus, a fast and reliable identification of these pathogens is very important for an immediate and successful medical treatment of such infections. Using the highly variable gene sequence encoding the flexible loop element of CCA-adding enzymes, we show that very closely related *Vibrio* and *Aspergillus* species can be discriminated at a much higher fidelity compared to approaches focusing on standard gene sequences in diagnostics.

## Results

One of the essential elements in the catalytic core of CCA-adding enzymes consists of a flexible loop region located between the conserved sequence motifs A and B (Fig. 1a). In contrast to other elements of the catalytic core, the amino acid composition of the loop reveals a remarkable sequence diversity. Hoffmeier et al*.* could show that this variety in the loop sequence correlates with the evolutionary distance between the corresponding organisms [15]. According to detailed sequence alignments, distant organisms exhibit great differences in their loop sequence, whereas the sequences of closely related organisms are more similar and can be summarized into loop families. Yet, the loop-encoding DNA sequences can be used for a reliable genotyping and are better suited for a robust species-specific identification than 16S rRNA analysis, a standard tool to identify and distinguish certain pathogenic bacteria [51].

To obtain species-specific sequences of the 16S rRNA, genomic DNA preparations of *V. alginolyticus*, *V. parahaemolyticus* and *V. vulnificus* were used for PCR-based amplification. As the first 527 base pairs were shown to have a distinction potential identical to the full-length sequence [52], we identified the 5′-terminus of the amplified 16S rRNA genes (850 nucleotides). The obtained sequences were identical to the corresponding entries found in the NCBI database (National Center for Biotechnology Information; http://www.ncbi.nlm.nih.gov/sutils/genom_table.cgi), verifying that the correct genes were amplified. In a corresponding alignment, characteristic point mutations were identified and their ratio between the compared species was calculated as the discriminatory potential (Table 1). Likewise, the gene section encoding the flexible loop region of the individual CCA-adding enzymes was sequenced and the number of base differences was determined.

**Table 1:** Discriminatory potential of the loop-encoding DNA sequence compared to 16S (*Vibrio*) or 18S rRNA gene (*Aspergillus*)

	16S rRNA gene			CCA-adding enzyme loop			Comparative potential
	Length [bp]	Number of specific mutations	%	Length [bp]	Number of specific mutations	%	%/%
*V. alginolyticus*	850	9	1.1	75	10	13.3	12.1
*V. parahaemolyticus*	850	1	0.1	75	8	10.7	107.0
*V. vulnificus*	850	42	4.9	75	21	28.0	5.7
Interspecific similarity	94.0–98.8 %			65.3–82.7 %			
	18S rRNA gene						
*A. fumigatus*	850	3	0.4	63	9	14.3	35.8
*A. niger*	850	1	0.1	63	10	15.9	159.0
*A. terreus*	850	4	0.5	63	7	11.1	22.2
Interspecific similarity	99.2–99.5 %			74.6–79.4 %			

The discriminatory potential is given as percentage sequence differences. The comparative potential is represented by the ratio of the discriminatory potentials. Interspecific similarities were calculated based on pairwise alignments in Fig. 1b. For the 16/18S rRNA gene, the first 850 positions were taken into account, whereas the flexible loop sequence was analyzed in full length. Although the loop sequence represents only 7.4 to 8.8 % in length compared to the analyzed rRNA gene region, the percentage values of mutations show a substantially higher discrimination potential of this sequence. The specific mutations within the indicated genes are unique and characteristic for the individual species

In addition to these bacterial species, representatives of pathogenic fungi were investigated as well. The corresponding gene sequences (18S rRNA gene, loop region of the CCA-adding enzyme gene) were analyzed in three closely related *Aspergillus* species, using genomic DNA of *A. fumigatus*, *A. niger* (ATCC 6275) and *A. terreus* (ATCC 10690). In all investigated 16S or 18S rRNA gene sequences, a discriminatory potential represented by percentage sequence differences from 0.1 to 1.1 % (*V. parahaemolyticus*, *V. alginolyticus*) and 0.1 to 0.5 (all tested *Aspergillus* species) was observed. Only the 16S rRNA gene of *V. vulnificus* exhibits a higher sequence deviation with 42 point mutations and deletions, leading to a ratio of 4.9 %. In all cases, a much higher sequence difference was observed for the gene region encoding the flexible loop of the corresponding CCA-adding enzymes. Here, mutation ratios between 10.7 and 13.3 % (*V. alginolyticus*, *V. parahaemolyticus*) and 11.1 and 15.9 % (*Aspergillus* species) were identified, whereas the discriminatory potential of the loop sequence from *V. vulnificus* reaches 28.0 % (Table 1). In this case, the comparative potential (represented by the ratio between specific mutations (in %) in the loop and the 16S rRNA sequences) is 5.7 times higher than that of the 16S rRNA analysis. For the other investigated organisms, the discriminatory potential is also higher for the loop sequence, ranging from a factor of 12.1 to 35.8. In the case of *V. parahaemolyticus* and *A. niger*, the first 850 nucleotides of the corresponding rRNA genes revealed only one characteristic point mutation, whereas the loop sequence presents 8 and 10 point mutations in a sequence of 75 and 63 nucleotides, respectively. Thus, the distinction potential is increased by factors 107.0 and 159.0 in these species.

The taxonomic resolution of an individual DNA sequence is defined by intra- and interspecific similarities [5]. These values are represented by the percentage discriminatory mutations in the compared versions of a specific sequence, either within strains of one species (intraspecific similarity) or within different species (interspecific similarity). Based on the sequence alignment in Fig. 1b, the interspecific similarity of the loop DNA sequences was obtained by a comparison of the individual *Vibrio* strains, leading to values between 65.3 and 82.7 % (Table 1). For the *Aspergillus* strains, 74.6 to 79.4 % were calculated. To determine the intraspecific similarity, the corresponding gene sequences of individual *Vibrio* and *Aspergillus* strains found in the NCBI database were investigated (Table 2). For *V. alginolyticus*, *V. parahaemolyticus* and *V. vulnificus*, the intraspecific sequence similarities have a range from 94.7 to 100 %, comparable to that of other *Vibrio*-specific markers like *toxR* or *rctB* [5]. In the case of *A. fumigatus*, *A. niger* and *A. terreus*, we observed a surprisingly high sequence identity. None of the strain-specific loop sequences showed any discriminating point mutation, resulting in an intraspecific similarity of 100 %.

**Table 2:** Intraspecific sequence similarities of selected *Vibrio* and *Aspergillus* species

Species	Number of strains	min. point mutations	max. point mutations	Similarity [%]
*V. alginolyticus*	8	0	1	98.7–100.0
*V. parahaemolyticus*	46	0	1	98.7–100.0
*V. vulnificus*	7	0	4	94.7–100.0
*A. fumigatus*	8	0	0	100.0
*A. niger*	7	0	0	100.0
*A. terreus*	3	0	0	100.0

DNA sequences of the flexible loop region from different strains were aligned and point mutations were identified, focusing on the strains with the least and the most mutations. The intraspecific similarity was calculated relative to the length of the respective loop sequence (63 and 75 bp, respectively)

**Fig. 2:**
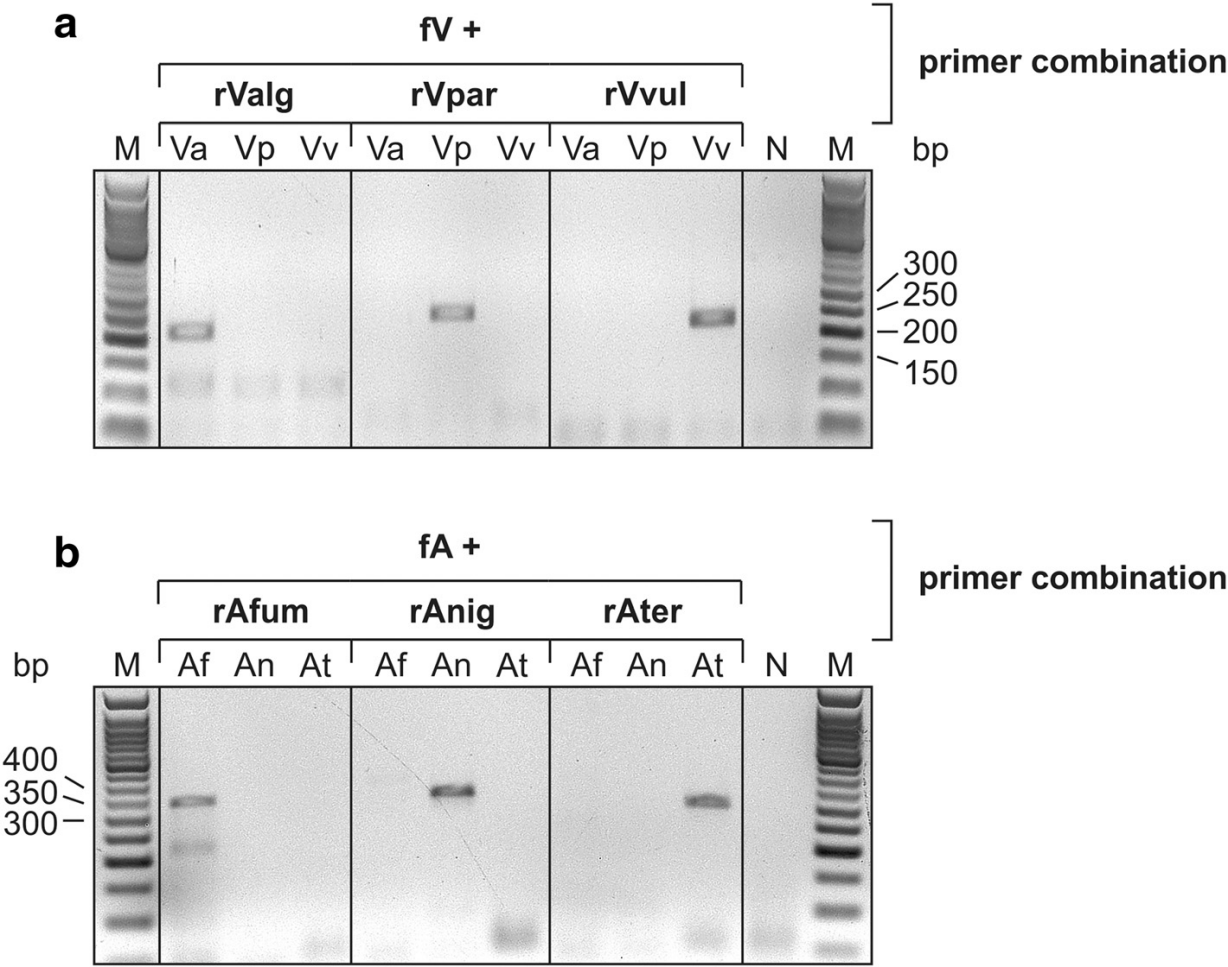
Genome-specific amplification of loop-encoding DNA sequences. **a**. A *Vibrio*-specific forward primer (fV) was used in combination with species-specific reverse primers rValg, rVpar and rVvul to selectively amplify the loop-encoding regions. 1.0 ng genomic DNA of *V. alginolyticus* (Va), *V. parahaemolyticus* (Vp) and *V. vulnificus* (Vv) was used as template. The respective PCR products have a length of 204, 245 and 231 bp, respectively. **b**. The corresponding gene region from 3.0 ng genomic DNA from *A. fumigatus*, *A. niger* and *A. terreus* was amplified with appropriate primers (fA with rAfum, rAnig or rAter), leading to PCR products of 333 (*A. fumigatus*, *A. niger*) and 301 bp (*A. terreus*). Reaction products were separated on a 2 % agarose gel and stained with ethidium bromide. N, PCR negative control. M, 50 bp DNA ladder (NEB)

Next, suitable and unique primer annealing regions in the flexible loop-coding DNA sequence were identified, allowing for a specific and highly selective amplification of the corresponding sequences. Sequence alignments revealed clusters of point mutations and presented segments suited for species-specific primer hybridization (Fig. 1). As single mismatches in the 3′-part of the primer sequence inhibit efficient annealing and, consequently, effective elongation and amplification, only annealing sites carrying at least two or three characteristic point mutations in the corresponding region were selected for each reverse primer. The individual forward primers are annealing in the region encoding motif A, which reveals a much higher DNA sequence homology than the flexible loop region. In a PCR reaction, all primer pairs were tested for selective amplification on the individual DNA preparations of the respective organisms. Agarose gel electrophoresis of the resulting PCR products indicated a highly specific amplification for the corresponding genomic DNA samples, leading to reaction products of 201 to 245 bp for the *Vibrio* species and 301 to 333 bp for the *Aspergillus* species (Fig. 2). In contrast, DNA from the other *Vibrio* or *Aspergillus* species did not lead to any detectable PCR signal. Hence, the bands in Fig. 2 represent highly specific amplification products for the tested individual prokaryotic and eukaryotic strains.

**Fig. 3:**
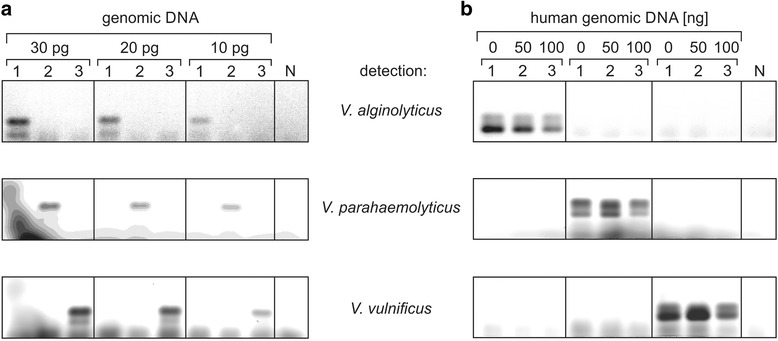
Multiplex PCR with individual fluorescence-labeled primers for different *Vibrio* strains. **a**. Species-specific amplification of the flexible loop-encoding DNA sequence. Indicated amounts of individual genomic DNA (1: *V. alginolyticus*, 2: *V. parahaemolyticus*, 3: *V. vulnificus*) were added to the primer mix. PCR products were visualized in the agarose gel by the different fluorescence of the species-specific primers. Down to 10 pg of each DNA sample were readily detected, without any cross reactivity with the other genomes. N, negative control. **b**. Human DNA does not interfere with the specific detection of *Vibrio* DNA. 0.1 ng of genomic DNA (1: *V. alginolyticus*, 2: *V. parahaemolyticus*, 3: *V. vulnificus*) were mixed with a 500 to 1,000-fold excess (50 and 100 ng) of human genomic DNA in a multiplex PCR and visualized as above. Compared to the positive control (0, no human DNA added), no additional bands appeared, indicating an exclusive and highly specific amplification of *Vibrio* DNA only. N, negative control with 50 ng of human genomic DNA

The high species-specificity in the amplification indicates that the flexible loop sequence has a high potential as a discriminatory marker in *Vibrio* or *Aspergillus* genotyping. To further simplify such an analysis, the performance of the individual primer combinations was tested in a multiplex PCR, allowing a simultaneous detection of individual strains. Reverse primers rValg, rVpar and rVvul, carrying 5′-terminal fluorophores ATTO 425, ATTO 532 and ATTO 633, respectively, were used in combination with the *Vibrio*-specific forward primer fV. The reaction products were separated on an agarose gel. Each fluorophore was excited by the appropriate wave length, and the specific emission signals showed a detection limit of 10 pg of DNA (Fig. 3a). The excitation and emission spectra of the selected fluorophores allow for a specific and distinct detection of the individually tested *Vibrio* sequences. The observed double bands in Fig. 3a are the consequence of a length heterogeneity of the labeled primers (data not shown) but not the result of unspecific hybridization during the amplification process.

Usually, patient samples do not only contain DNA from the pathogen but carry an excess amount of endogenous human genomic DNA. As the human genome also encodes for a related CCA-adding enzyme, it is important that the genotyping procedure strongly discriminates against such endogenous contaminations. Hence, 0.1 ng of *Vibrio* DNA was mixed with an increasing amount of human genomic DNA, leading to a 1,000-fold excess of human gene sequences. With such contaminated material, a multiplex PCR containing the above mentioned primer combinations was performed and the reaction products were separated on an agarose gel (Fig. 3b). The resulting fluorescent signals indicate that the excess of human DNA has only a minor effect on the amplification efficiency of the *Vibrio* sequences, while no unspecific reaction products resulting from hybridization to the human DNA were observed. Nevertheless, we have designed a human-specific blocking oligonucleotide carrying a C3 spacer at the 3′ position that efficiently inhibits the amplification of the human loop-encoding sequence, while it does not interfere with PCR-based detection of bacterial sequences (Additional file 1: Figure S1) [53]. Hence, if needed, this blocking oligonucleotide can be added to the PCR reaction in order to enhance the detection specificity.

Taken together, these results confirm that the DNA sequence encoding the flexible loop from CCA-adding enzymes represents a unique and highly specific amplification target that allows a fast and reliable genotyping of different *Vibrio* or *Aspergillus* infections.

## Discussion

Infections caused by pathogenic microorganisms represent a widespread threat of human health. Especially the incidences of foodborne diseases and invasive fungal infections caused by *Vibrio* and *Aspergillus* species are increasing every year. Representatives of these two genera are able to thrive on a broad spectrum of substrates and environmental conditions, leading to their world-wide distribution that is even enhanced by climate changes. Especially the three non-cholera *Vibrio* species *V. alginolyticus*, *V. parahaemolyticus* and *V. vulnificus*, all thriving in coastal and estuarine waters, are benefiting from global warming and increasing surface temperatures of lakes and seas all over the world [39, 42, 43, 54, 55]. Hence, the development of fast and reliable identification methods for such human pathogens is urgently needed. Since many microbiological analyses were replaced by faster and more sensitive PCR methods, many specific gene sequences were identified that allow for a distinction between closely related species of the same genus. For such species-specific identification, the analysis of 16S and 18S rRNA gene sequences represents a widely used standard approach [2, 51, 56–58]. However, in the case of *V. alginolyticus*, *V. parahaemolyticus* and *V. vulnificus* as well as *A. fumigatus*, *A. niger* and *A. terreus*, the distinctive potential of the corresponding ribosomal DNA sequences is rather low, ranging from 0.1 to 4.9 % (Table 1). The high similarities in the 16S rRNA gene sequence were already described by Kwok et al*.*, who observed a sequence identity of 99 % between *V. alginolyticus* and *V. parahaemolyticus* and a 95 % between *V. alginolyticus* and *V. vulnificus*, as well as between *V. parahaemolyticus* and *V. vulnificus* [59]. Likewise, other studies revealed that rRNA sequence homologies between *V. parahaemolyticus* and related species are so high that this gene is not suited for identification approaches [60, 61].

To overcome these limitations, more specific template sequences for reliable genotyping were established in the last years. Especially for the discrimination of *Vibrio* species, pathogenicity markers like thermolabile hemolysin (*tlh*), thermostable direct hemolysin (*tdh*) and thermostable direct hemolysin-related hemolysin (*trh*) are analyzed [62–65]. A very useful target sequence to identify different *Vibrio* species is the *toxR* gene, a toxicity-related transcriptional regulator originally found in *V. cholerae* [5, 66, 67]. Combined in multiplex PCR or multi locus sequence typing approaches, these *Vibrio*-specific target genes are powerful tools for a solid identification procedure. However, they are restricted to a small pool of organisms within a genus, showing no distribution in other human-relevant pathogens. Studies on more ubiquitous gene sequences, like the B subunit of the bacterial DNA gyrase (*gyrB*), the *hsp60* or the *rpoB* gene, revealed differences ranging from 3.2 to 20.0 % between *V. alginolyticus*, *V. parahaemolyticus* and *V. vulnificus* [59, 68, 69]. In 2010, Pascual et al*.* published a multi locus sequence analysis of several closely related *Vibrio* species, calculating the intra- and interspecific sequence similarities of the most promising genotyping sequences. Intraspecific sequence similarities were ranging from 98.8–100 % (16S rRNA gene), 92.7–100 % (*recA*), 93.7–100 % (*pyrH*), 95.6–100 % (*rpoD*), 86.8–100 % (*gyrB*), 85.6–100 % (*rctB*) and 77.2–100 % (*toxR*), with interspecific similarities of 97.6–99.9 % (16S rRNA gene), 87.9–99.9 % (*recA*), 86.4–97.8 % (*pyrH*), 79.1–96.0 % (*rpoD*), 83.1–99.5 % (*gyrB*), 74.3–92.7 % (*rctB*) and 33.8–72.5 % (*toxR*) [5]. According to these values, the best taxonomic resolution is given by the sequences of *rctB*, *rpoD* and *toxR*.

**Fig. 4:**
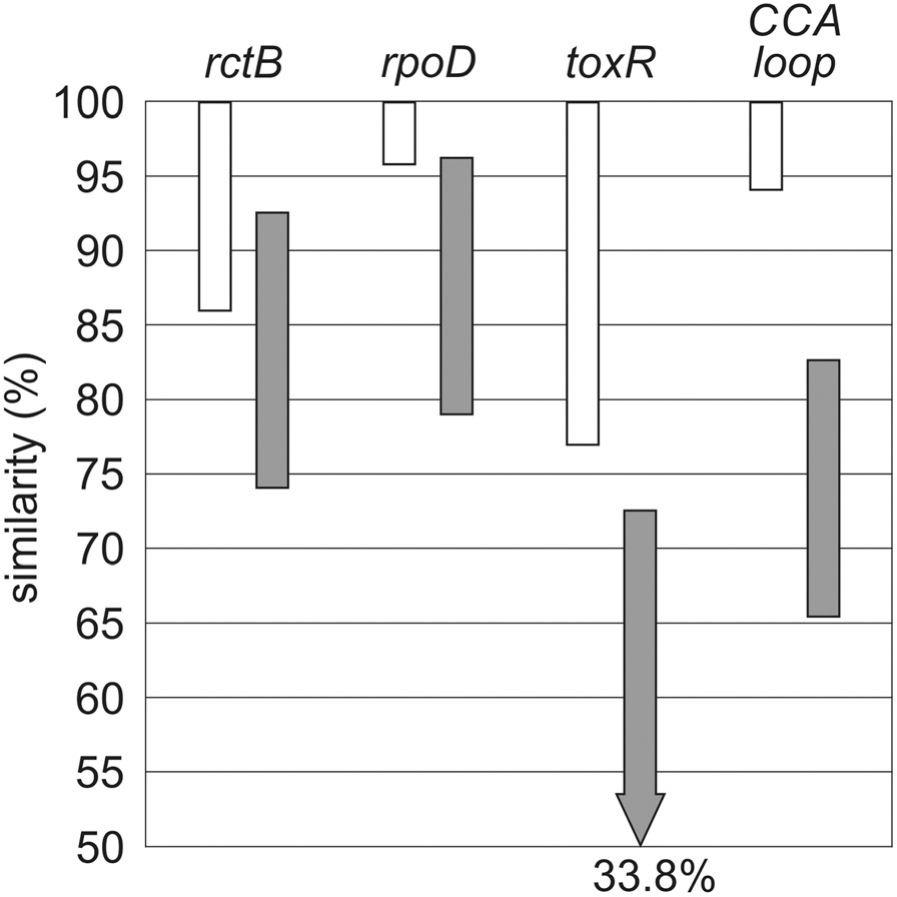
Taxonomic resolution of *Vibrio*-specific sequence markers. According to Pascual et al., the ranges of intraspecific (*open bars*) versus interspecific (*filled bars*) similarities (%) are indicated [5]. In contrast to the frequently used marker genes *rctB* and *rpoD*, the gene sequence encoding the loop sequence of the CCA-adding enzyme shows a superb resolution, comparable to that of *toxR*

Compared to these established *Vibrio*-discriminating sequences, the region encoding the flexible loop of the CCA-adding enzyme shows an intraspecific similarity of 94.7 to 100 %, while the interspecific sequence similarities lie between 65.3 and 82.7 % (Fig. 4). Furthermore, in 46 strains of *V. parahaemolyticus*, only a single point mutation was observed in two strains, corresponding to an intraspecific similarity of 98.7 to 100 % (Table 2). For *V. alginolyticus* and *V. vulnificus*, comparably high values (98.7–100 % and 94.7–100 %) were obtained, indicating that the taxonomic resolution (defined by the gap between the maximum intraspecific distance and the minimum interspecific distance [5]) outperforms the usual *Vibrio* marker genes *rctB* and *rpoD*, while it is nearly as distinctive as the *toxR* gene (Fig. 4). A striking exception is *V. alginolyticus* strain E0666, where nine intraspecific point mutations were detected in the loop-encoding region, corresponding to an intraspecific similarity of 88.0 %. However, the *toxR* sequence of the same strain shows a comparable intraspecific similarity of 87.6 %, as this gene also carries an unexpected high number of characteristic point mutations (109 in 879 bp). As all other investigated *V. alginolyticus* genomes showed sequence similarities of 98.7 % in both *toxR* and loop sequence, E0666 was not included in our analysis, as it is very likely that it does not represent a true *V. alginolyticus* strain. Nevertheless, even when this strain is included, the intraspecific similarity of *V. alginolyticus* would be between 88.0 and 100 %, again comparable to that of *toxR*.

Taken together, these classification parameters indicate that the flexible loop region of CCA-adding enzymes has an identification potential similar to that of established genetic *Vibrio* markers, although its sequence with a length of 75 bp is much shorter than that of the main discriminatory regions of *toxR* (477 bp), *rctB* (591 bp) and *rpoD* (780) [5]. Furthermore, while *toxR* and *rctB* markers can only be used for *Vibrio* genotyping, CCA-adding enzyme genes with varying flexible loop regions are found in all bacteria and eukaryotes, indicating that the loop sequence has the potential as a genotyping marker for a wide range of different pathogens [15].

This is further supported by our findings concerning the genotyping of individual *Aspergillus* species. Here, the discriminatory potential of the loop sequences of *A. fumigatus*, *A. niger* and *A. terreus* is even higher, with interspecific similarities between 74.6 and 79.4 % compared to 99.2–99.5 % in the 18S rRNA gene sequences (Table 1, Fig. 1b). In addition, no intraspecific point mutations were observed (Table 2), leading to an excellent taxonomic resolution. Yet, 18S rRNA sequence analysis is frequently used for *Aspergillus* genotyping [2, 70, 71]. Alternatively, random amplification of polymorphic DNA (RAPD) and restriction fragment length polymorphism PCR (RFLP-PCR) are used, where the internal transcribed spacer regions between 18S, 5.8S and 28S rRNA genes are investigated [50, 72–74]. Both methods are suitable for a reliable identification of *Aspergillus* species, but the correct interpretation of the resulting complex DNA fragment patterns is difficult [73]. Furthermore, these methods cannot be used for analyzing several species within a single multiplex PCR, as it was done in this study. In contrast, the flexible loop-encoding region of the gene for CCA-adding enzyme is much better suited for identification of pathogenic microorganisms (in single or multiplex PCR), fulfilling the criteria for a successful application in genotyping [4]. First, as the CCA-adding enzyme represents an essential protein, the corresponding gene is ubiquitously distributed in the kingdoms of Bacteria and Eukaryotes [75, 76]. Second, to allow a specific sequence analysis, the target gene must be unique within a genome, without closely related paralogs. Bacterial and eukaryotic CCA-adding enzymes are closely related to bacterial poly(A) polymerases, sharing the same conserved core motifs [77, 78]. However, the flexible loop element of the poly(A) polymerase has a very unique sequence composition, showing no similarity to the loop sequence of CCA-adding enzymes [11]. Furthermore, in several bacterial species, the CCA-adding activity is shared between two closely related enzymes, where the first catalyzes the addition of two C residues (CC-adding enzyme), while the second one adds the terminal A (A-adding enzyme) [79–81]. Yet, such a gene constellation still allows for a specific species identification, as CC-adding enzymes have lost the loop region due to a deletion of the corresponding coding region [13]. In contrast, A-adding enzymes carry a loop sequence. However, this sequence shows the same discriminatory potential as in CCA-adding enzymes, so that the loop region in A-adding enzymes can also be used for genotyping. Third, the gene sequence has to be long enough to contain sufficient phylogenetic information, and, on the other hand, short enough for fast sequence analysis using a minimal set of primers. With a length of 63–75 bp, as found in *Vibrio* and *Aspergillus* species as well as in other organisms, the loop-encoding region is indeed very short. Nevertheless, our data indicate that it contains a high number of unique and characteristic sequence differences for a species-specific amplification and identification.

## Conclusions

Taken together, the DNA sequence encoding the flexible loop region of CCA-adding enzymes shows all features required for a highly discriminating gene marker in pathogenic microorganisms. This is supported by the presented results, where closely related pro- and eukaryotic pathogens could be easily discriminated, in contrast to most of the established diagnostic markers. Although in the case of *Aspergillus*, no loop sequence information of type strains is available, the data clearly show that this DNA marker is also very useful for eukaryotic pathogens. Combined with other sequences with high discriminatory potential, this new marker will contribute to a superb and highly reliable diagnostic procedure in infectology and systematic microbiology. Furthermore, the loop sequence can be a useful diagnostic tool in food hygiene analysis or in the detection of specific microbial species in so far unidentifiable metagenome DNA sequences.

## Methods

### Microbial strains and DNA extraction

Genomic DNA of *V. alginolyticus* (ATCC 17749), *V. parahaemolyticus* (ATCC 17802) and *V. vulnificus* (ATCC 27562) was obtained from the Leibniz Institute DSMZ - German Collection of Microorganisms and Cell Cultures (Braunschweig, Germany). *A niger* and *A. terreus* were obtained as freeze-dried cultures from the same institute. P. Zipfel (Leibniz Institute for Natural Product Research and Infection Biology - Hans Knöll Institute, Jena, Germany) provided the genomic DNA of *A. fumigatus*. The freeze-dried cultures were handled as directed and dissolved in 40 % glycerol for storage at −20 °C. 50 ml Sabouraud dextrose broth were inoculated with 200 μl of the glycerol stock and incubated for 24 h at 37 °C and 200 rpm. After a centrifugation step, the pellet was resuspended in DNA extraction buffer containing 0.7 M NaCl, 0.1 M Tris–HCl (pH 7.5), 50 mM EDTA and 1 % SDS (w/v). Cells were lysed in a FastPrep®-24 system (MP Biomedicals, Heidelberg, Germany), using lysing matrix C at 6.0 m/s for 30 s. After a second centrifugation step, the supernatant was incubated for 5 min at room temperature and mixed with 5 ml 24:1 chloroform/isoamyl alcohol for 30 min on ice. The DNA in the supernatant was precipitated with 100 % ethanol. After centrifugation, the pellet was washed with 70 % ethanol, air dried, and dissolved in 50 μl double-distilled water. DNA concentrations were determined on a NanoDrop™ 1000 spectrophotometer (Thermo Fisher Scientific, Braunschweig, Germany).

Gene sequences of the individual CCA-adding enzymes were obtained from the National Center for Biotechnology Information (NCBI) database (Additional file 2: Table S1). The loop-encoding sequences were identified by aligning the corresponding DNA sequence between conserved core motifs A and B. 16S and 18S rRNA gene regions were sequenced on an ABI Prism 3700 automated sequencer (Amersham Biosciences). Accession numbers of further 16/18S rRNA genes used for interspecific alignments are summarized in Additional file 3: Table S2.

### Oligonucleotide primers

For the specific amplification of loop-encoding DNA sequences, a universal forward primer was used that recognizes the conserved DNA sequence of motif A of the CCA-adding enzyme gene of *Vibrio* (fV) or *Aspergillus* species (fA). The reverse primers hybridize to the individual loop-encoding DNA sequences and are therefore highly specific for the individual species. Annealing regions for reverse primers are shown in Fig. 1b. Primer sequences including fluorescent 5′-labels (ATTO 425, ATTO 532, ATTO 633; biomers, Ulm, Germany) are summarized in Table 3.

**Table 3:** Sequences of primers used in this study

Primer	Sequence	Description	5′-label	abs (nm)	em [nm]
fV	5′- GTAGGTGGCGCAGTTCGAG -3′ 5′- GTGGGTGGAGCGGTAAGAG -3′	Degenerated forward primer for motif A region of *V. alginolyticus*/*V. parahaemolyticus* (sequence 1) and *V. vulnificus* (sequence 2)			
rValg	5′- ACCAGTGTAACCAGAACCTGAC -3′	Reverse primer for loop sequence of *V. alginolyticus*	ATTO 425	436	484
rVpar	5′- TCCTCTTCTAGCGTTACGCTTG -3′	Reverse primer for loop sequence of *V. parahaemolyticus*	ATTO 532	532	553
rVvul	5′- CACATCTGGGGAGAAAAAGCAC -3′	Reverse primer for loop sequence of *V. vulnificus*	ATTO 633	629	657
fA	5′- GGGTGAGGGACAAGCTG -3′ 5′- GGGTGCGGGACAAGCTG -3′	Degenerated forward primer for motif A region of *A. fumigatus*/*A. niger* (sequence 1) and *A. terreus* (sequence 2)			
rAfum	5′- CATCTTCTTCGGCGGTG -3′	Reverse primer for loop sequence of *A. fumigatus*			
rAnig	5′- CGTCTTCCTGGGCTGTTC -3′	Reverse primer for loop sequence of *A. niger*			
rAter	5′- GGATTCCGACTATCATCCGTATAG -3′	Reverse primer for loop sequence of *A. terreus*			

### Standard and multiplex PCR amplifications

PCR was carried out in a volume of 20 μl of 20 mM Tris/HCl (pH 8.4), 50 mM KCl, 2 mM Mg Cl_2_, 150 μM dNTPs, 0.3 μM forward and reverse primers, 1 % DMSO, 1 U *Taq* DNA polymerase (NEB) and 0.1 to 3.0 ng of genomic DNA. An initial denaturation for 3 min at 96 °C was followed by 40 cycles of denaturation (96 °C, 60–90 s), primer annealing (53/59 °C, 60 s) and elongation (68 °C, 30/60 s). A final elongation was performed at 68 °C for 5 min. To improve the hybridization specificity in some of the reactions, a touch-down PCR was performed with temperature gradient for primer annealing from 60 °C to 53 °C within the first 10 cycles.

### Detection of PCR products and imaging

PCR products were separated on an agarose gel and visualized by ethidium bromide staining. Fluorescent PCR products were detected by scanning the agarose gel in a *Typhoon 9410 Variable Mode Imager* using suitable laser-filter combinations. The absorption and emission maxima of the fluorescent labels are presented in Table 3. Resulting images were saved as tagged image files (tif).

### Sequence alignments

DNA sequences encoding the individual loop or rRNA sequences were aligned using Clustal Omega (http://www.ebi.ac.uk/Tools/msa/clustalo/) with default parameters [82].

## Additional files

Additional file 1: Figure S1. A 3′-blocked oligonucleotide efficiently inhibits the amplification of human loop-encoding DNA. **A.** The blocking oligonucleotide (black) recognizes the human loop sequence (red) and part of the upstream located region encoding motif A (green). This interferes with binding of the PCR forward primer (green), and, consequently, amplification of the human sequence. The 3′- end of this oligonucleotide is blocked by a C3 spacer (x). **B.** Increasing ratios of blocking oligonucleotide versus forward primer inhibit amplification of the human sequence, while the bacterial sequence amplification remains unaffected. Hence, the blocking oligonucleotide can increase the selective amplification of pathogenic loop sequences in the PCR reaction. N, PCR negative control; M, 50 bp DNA ladder (NEB). (DOCX 4696 kb) Additional file 2: Table S1. Strains used for analysis of intraspecific similarities in the gene region encoding the loop sequence. Accession numbers indicate genome sequences of the corresponding strains. (DOCX 19 kb) Additional file 3: Table S2. Strains used for interspecific 16 or 18S rRNA gene sequence alignments. Accession numbers are indicating the 16/18S rRNA gene sequences of the corresponding strains. (DOCX 15 kb)
