# Erratum to: Genotyping bacterial and fungal pathogens using sequence variation in the gene for the CCA-adding enzyme

**DOI:** 10.1186/s12866-016-0841-1

**Published:** 2016-09-21

**Authors:** Paul Franz, Heike Betat, Mario Mörl

**Affiliations:** Institute for Biochemistry, Leipzig University, Brüderstr. 34, 04103 Leipzig, Germany

## Erratum:

Unfortunately, the original version of this article [1] contained an error, Fig. 3b was incorrectly labeled.

In regards to Fig. 3b, for the analysis of a possible impact of human DNA in the *Vibrio*-specific PCR amplification of loop-encoding DNA sequences, the individual lanes with admixture of 0, 50 or 100 ng of human DNA contained only DNA of one of the individual *Vibrio* strains. Hence, the labels 1, 2 and 3 (indicating different *Vibrio* strains) are erroneous.

A corrected version and legend for Fig. 3 is presented below:

**Fig. 3:**
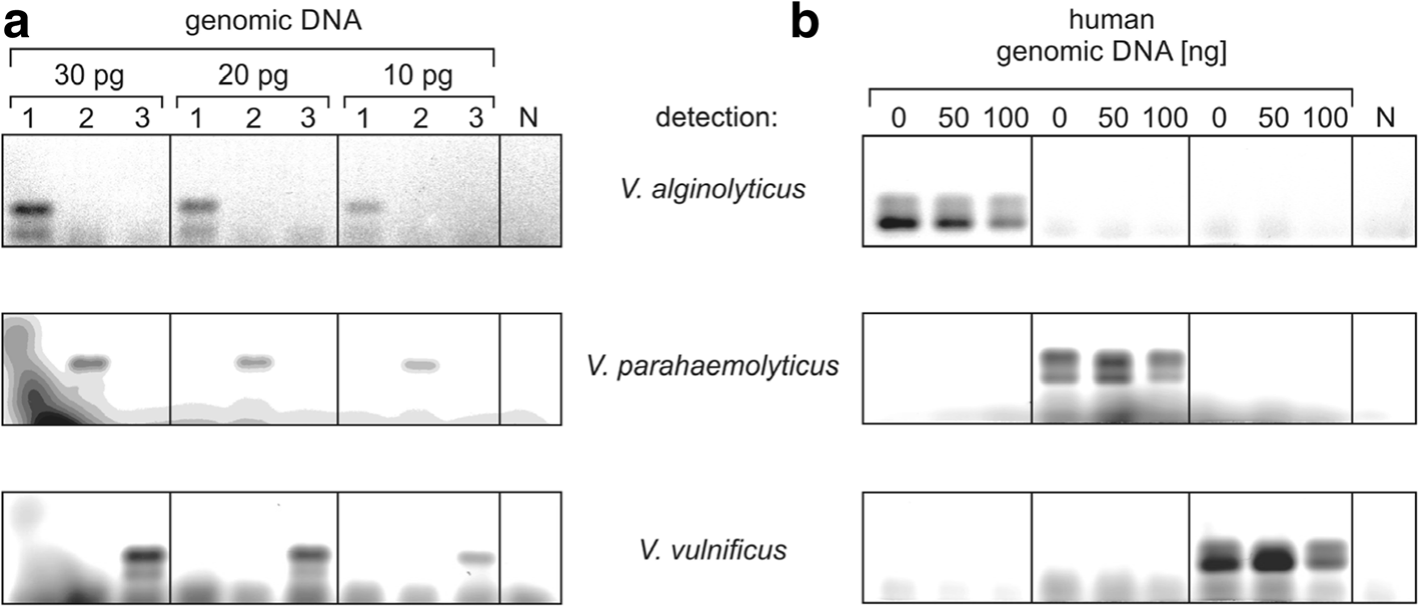
Multiplex PCR with individual fluorescence-labeled primers for different *Vibrio* strains. **a.** Species-specific amplification of the flexible loop-encoding DNA sequence. Indicated amounts of individual genomic DNA (1: *V. alginolyticus*, 2: *V. parahaemolyticus*, 3: *V. vulnificus*) were added to the primer mix. PCR products were visualized in the agarose gel by the different fluorescence of the species-specific primers. Down to 10 pg of each DNA sample were readily detected, without any cross reactivity with the other genomes. N, negative control. **b.** Human DNA does not interfere with the specific detection of *Vibrio* DNA. 0.1 ng of genomic DNA of the indicated *Vibrio* strains were mixed with a 500 to 1000-fold excess (50 and 100 ng) of human genomic DNA in a multiplex PCR and visualized as above. Compared to the positive control (0, no human DNA added), no additional bands appeared, indicating an exclusive and highly specific amplification of *Vibrio* DNA only. N, negative control with 50 ng of human genomic DNA
