# Supplementary material for: Genotyping bacterial and fungal pathogens using sequence variation in the gene for the CCA-adding enzyme
Source: BMC Microbiol. 2016 Mar 18;16:47. doi: 10.1186/s12866-016-0670-2 (PMC4797355; doi:10.1186/s12866-016-0670-2)
Supplement: Additional file 1: Figure S1. — A 3′-blocked oligonucleotide efficiently inhibits the amplification of human loop-encoding DNA. A. The blocking oligonucleotide (black) recognizes the human loop sequence (red) and part of the upstream located region encoding motif A (green). This interferes with binding of the PCR forward primer (green), and, consequently, amplification of the human sequence. The 3′- end of this oligonucleotide is blocked by a C3 spacer (x). B. Increasing ratios of blocking oligonucleotide versus forward primer inhibit amplification of the human sequence, while the bacterial sequence amplification remains unaffected. Hence, the blocking oligonucleotide can increase the selective amplification of pathogenic loop sequences in the PCR reaction. N, PCR negative control; M, 50 bp DNA ladder (NEB). (DOCX 4696 kb) [file 12866_2016_670_MOESM1_ESM.docx]

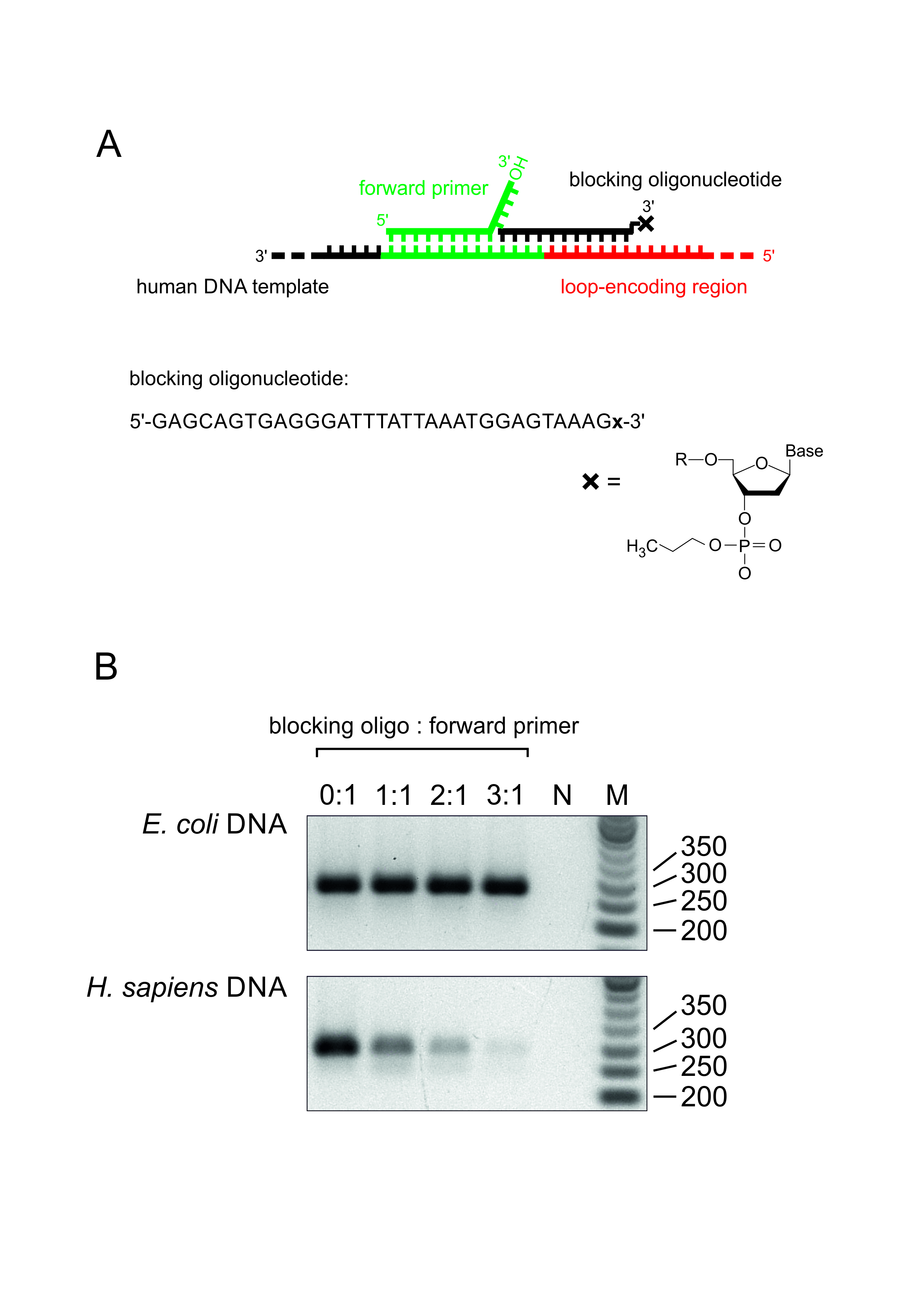


**Additional Figure 1:** A 3’-blocked oligonucleotide efficiently inhibits the amplification of human loop-encoding DNA. A. The blocking oligonucleotide (black) recognizes the human loop sequence (red) and part of the upstream located region encoding motif A (green). This interferes with binding of the PCR forward primer (green), and, consequently, amplification of the human sequence. The 3’end of this oligonucleotide is blocked by a C3 spacer (x). B. Increasing ratios of blocking oligonucleotide versus forward primer inhibit amplification of the human sequence, while the bacterial sequence amplification remains unaffected. Hence, the blocking oligonucleotide can increase the selective amplification of pathogenic loop sequences in the PCR reaction. N, negative control; M, 50 bp DNA ladder (NEB).
