# Supplementary material for: Genotyping bacterial and fungal pathogens using sequence variation in the gene for the CCA-adding enzyme
Source: BMC Microbiol. 2016 Mar 18;16:47. doi: 10.1186/s12866-016-0670-2 (PMC4797355; doi:10.1186/s12866-016-0670-2)
Supplement: Additional file 2: Table S1. — Strains used for analysis of intraspecific similarities in the gene region encoding the loop sequence. Accession numbers indicate genome sequences of the corresponding strains. (DOCX 19 kb) [file 12866_2016_670_MOESM2_ESM.docx]

**Additional Table 1:** Strains used for analysis of intraspecific similarities in the gene region encoding the loop sequence. Accession numbers indicate genome sequences of the corresponding strains.

| species | strain | accession number |
| --- | --- | --- |
| *Vibrio alginolyticus* | 12G01 | AAPS01000043.1 |
|  | 40B | ACZB01000059.1 |
|  | ATCC 17749 | CP006718.1 |
|  | ATCC 33787 | CP013484.1 |
|  | BSW8 | NZ_JXIN01000004.1 |
|  | BSW15 | NZ_JXIO01000009.1 |
|  | EPGS | NZ_JOKY01000009.1 |
|  | UCD-32C | NZ_LJTF01000002.1 |
| *Vibrio parahaemolyticus* | 50 | AZGT01000005.1 |
|  | 605 | AZGU01000003.1 |
|  | 861 | AZGV01000044.1 |
|  | 949 | AVPV01000004.1 |
|  | 3256 | AZGS01000026.1 |
|  | 3259 | AVOL01000005.1 |
|  | 10290 | AVOH01000008.1 |
|  | 10296 | AYSP01000127.1 |
|  | 12310 | AYXP01000028.1 |
|  | 12315 | AOPF01000052.1 |
|  | 10329 | AFBW01000018.1 |
|  | 970107 | AZIQ01000014.1 |
|  | AN-5034 | ACFO01000016.1 |
|  | AQ3810 | AAWQ01000157.1 |
|  | AQ4037 | ACFN01000123.1 |
|  | ATCC 17802 | sequenced |
|  | B-265 | AZKN01000014.1 |
|  | BB22OP | NC_019955.1 |
|  | CDC_K4557 | CP006008 |
|  | EKP-008 | AZMN01000136.1 |
|  | EKP-021 | AZNA01000217.1 |
|  | EKP-026 | JABV01000017.1 |
|  | FDA_R31 | NC_021847.1 |
|  | IDH02189 | JAHD01000019.1 |
|  | IDH02640 | JAIH01000036.1 |
|  | K5030 | ACKB01000016.1 |
|  | NIHCB0603 | AVOM01000003.1 |
|  | NIHCB0757 | AVPX01000004.1 |
|  | PCV08-7 | AVON01000009.1 |
|  | Peru-288 | JAII01000021.1 |
|  | Peru-466 | ACFM01000033.1 |
|  | RIMD 2210633 | NP_796793 |
|  | SBR10290 | JAIJ01000048.1 |
|  | UCM-V493 | NZ_CP007004.1 |
|  | V14/01 | JALG01000017.1 |
|  | V-223/04 | JALH01000709.1 |
|  | VP2007-007 | JALI01000398.1 |
|  | VP2007-095 | AVOI01000015.1 |
|  | VP-48 | JDFK01000039.1 |
|  | VP232 | AVOJ01000045.1 |
|  | VP250 | AVOK01000001.1 |
|  | VPCR-2009 | JDFL01000085.1 |
|  | VPCR-2010 | AVPW01000029.1 |
|  | VPTS-2009 | JDFM01000021.1 |
|  | VPTS-2010 | JDFN01000318.1 |
|  | VPTS-2010_2 | JDFO01000324.1 |
| *Vibrio vulnificus* | 93U204 | NZ_CP009261.1 |
|  | ATCC 27562 | sequenced |
|  | BAA87 | JDSE01000001.1 |
|  | CMCP6 | AAO09136.3 |
|  | MO6-24/O | CP002469.1 |
|  | VVyb1(BT3) | NZ_CM001799.1 |
|  | YJ016 | NC_005139.1 |
| *Aspergillus fumigatus* | A1163 | DS499595.1 |
|  | AF210 | PRJNA52783 |
|  | Af293 | XM_750910.2 |
|  | B5856 | PRJNA67101 |
|  | CEA10 | PRJNA258513 |
|  | **not known** | sequenced |
|  | var. RP-2014 | JHOI01000623.1 |
|  | Z5 | AZZA01000100.1 |
| *Aspergillus niger* | ATCC 1015 | ACJE01000017.1 |
|  | ATCC 6275 | sequenced |
|  | ATCC 10864 | PRJNA300350 |
|  | ATCC 13496 | PRJNA209543 |
|  | CBS 513.88 | XM_001402189.2 |
|  | N402 | PRJNA250529 |
|  | SH-2 | AUZU01000041.1 |
| *Aspergillus terreus* | ATCC 10690 | sequenced |
|  | LYT10 | PRJNA296012 |
|  | NIH2624 | XM_001211442.1 |
