# Supplementary material for: Genotyping bacterial and fungal pathogens using sequence variation in the gene for the CCA-adding enzyme
Source: BMC Microbiol. 2016 Mar 18;16:47. doi: 10.1186/s12866-016-0670-2 (PMC4797355; doi:10.1186/s12866-016-0670-2)
Supplement: Additional file 3: Table S2. — Strains used for interspecific 16 or 18S rRNA gene sequence alignments. Accession numbers are indicating the 16/18S rRNA gene sequences of the corresponding strains. (DOCX 15 kb) [file 12866_2016_670_MOESM3_ESM.docx]

**Additional Table 2:** Strains used for interspecific 16 or 18S rRNA gene sequence alignments. Accession numbers are indicating the 16/18S rRNA gene sequences of the corresponding strains.

| species | strain | accession number |
| --- | --- | --- |
| *Vibrio alginolyticus* | ATCC 17749 | NR_119049.1 |
| *Vibrio parahaemolyticus* | ATCC 17802 | NR_114630.1 |
| *Vibrio vulnificus* | ATCC 27562 | NR_119061.1 |
| *Aspergillus fumigatus* | not known | AB008401.1 |
| *Aspergillus niger* | ATCC 6275 | D63697.1 |
| *Aspergillus terreus* | ATCC 10690 | AB008409.1 |
